# Plasma microRNA ratios associated with breast cancer detection in a nested case–control study from a mammography screening cohort

**DOI:** 10.1038/s41598-023-38886-0

**Published:** 2023-07-25

**Authors:** Giovanna Chiorino, Elisabetta Petracci, Emir Sehovic, Ilaria Gregnanin, Elisa Camussi, Maurizia Mello-Grand, Paola Ostano, Emilia Riggi, Viviana Vergini, Alessia Russo, Enrico Berrino, Andrea Ortale, Francesca Garena, Tiziana Venesio, Federica Gallo, Elisabetta Favettini, Alfonso Frigerio, Giuseppe Matullo, Nereo Segnan, Livia Giordano

**Affiliations:** 1 Cancer Genomics Lab, Fondazione Edo ed Elvo Tempia, Via Malta 3, 13900 Biella, Italy; 2 Unit of Biostatistics and Clinical Trials, IRCCS Istituto Romagnolo per lo Studio dei Tumori (IRST) “Dino Amadori”, Meldola, Italy; 3 Department of Life Sciences and Systems Biology, University of Turin, Turin, Italy; 4 SSD Epidemiologia Screening, CPO-AOU Città della Salute e della Scienza di Torino, Via Camillo Benso Di Cavour 31, 10123 Turin, Italy; 5 Department of Medical Sciences, University of Turin, Turin, Italy; 6 Pathology Unit, Candiolo Cancer Institute, FPO IRCCS, Candiolo, Italy; 7 Epidemiology Unit, Staff Health Direction, Local Health Authority 1 of Cuneo, Cuneo, Italy; 8 Diagnostic Radiology Unit, Nuovo Ospedale Degli Infermi, Ponderano, Italy

**Keywords:** Computational biology and bioinformatics, Molecular biology, Biomarkers, Risk factors, Breast cancer, Cancer prevention, Cancer screening, Tumour biomarkers, Cancer, Cancer

## Abstract

Mammographic breast cancer screening is effective in reducing breast cancer mortality. Nevertheless, several limitations are known. Therefore, developing an alternative or complementary non-invasive tool capable of increasing the accuracy of the screening process is highly desirable. The objective of this study was to identify circulating microRNA (miRs) ratios associated with BC in women attending mammography screening. A nested case–control study was conducted within the ANDROMEDA cohort (women of age 46–67 attending BC screening). Pre-diagnostic plasma samples, information on life-styles and common BC risk factors were collected. Small-RNA sequencing was carried out on plasma samples from 65 cases and 66 controls. miR ratios associated with BC were selected by two-sample Wilcoxon test and lasso logistic regression. Subsequent assessment by RT-qPCR of the miRs contained in the selected miR ratios was carried out as a platform validation. To identify the most promising biomarkers, penalised logistic regression was further applied to candidate miR ratios alone, or in combination with non-molecular factors. Small-RNA sequencing yielded 20 candidate miR ratios associated with BC, which were further assessed by RT-qPCR. In the resulting model, penalised logistic regression selected seven miR ratios (miR-199a-3p_let-7a-5p, miR-26b-5p_miR-142-5p, let-7b-5p_miR-19b-3p, miR-101-3p_miR-19b-3p, miR-93-5p_miR-19b-3p, let-7a-5p_miR-22-3p and miR-21-5p_miR-23a-3p), together with body mass index (BMI), menopausal status (MS), the interaction term BMI * MS, life-style score and breast density. The ROC AUC of the model was 0.79 with a sensitivity and specificity of 71.9% and 76.6%, respectively. We identified biomarkers potentially useful for BC screening measured through a widespread and low-cost technique. This is the first study reporting circulating miRs for BC detection in a screening setting. Validation in a wider sample is warranted.

Trial registration: The Andromeda prospective cohort study protocol was retrospectively registered on 27-11-2015 (NCT02618538).

## Introduction

Breast cancer (BC) is the most commonly diagnosed cancer in females (2,261,419 new cases, 11.7% of all cancer sites) and the leading cause of women cancer death worldwide (684,996 deaths, 6.9% of all sites) as reported in the Global Cancer Statistics report for 2020^1^.

Currently, the primary screening tool for early detection is mammography. Although it is demonstrated to be effective in reducing cancer mortality (around 15% in women younger than 50 years, and between 14 and 23% in older women), it does have certain limitations including interval cancers, false positive rate, overdiagnosis, radiation exposure and inflexible scheduling^2,3^. Notably, the updated European Breast Screening Guidelines of the European Commission Initiative for Screening and Diagnosis strongly recommended biennial mammography screening in the context of an organised program for women 50–69 years. Organised mammography screening is suggested, but with a conditional recommendation, also for younger (45–49 years) and older women (70–74 years), while screening interval (biennial or triennial) in these age ranges is still under debate^4^.

Lately, age extensions, diverse imaging technologies and combinations of different risk factors have been considered for optimizing BC screening protocols. BC risk is a composite measure, including the contribution of reproductive history (i.e. menarche, menopause, age at first pregnancy), family history, previous breast biopsies, prior chest irradiation, and breast density^5,6^. The state-of-the-art BC risk algorithms also include (epi)genetic biomarkers, such as single nucleotide polymorphisms (SNPs) or microRNAs (miRs)^6–8^. miRs are a class of small non-coding RNA molecules which function as negative regulators of gene expression by directing specific mRNA cleavage or translational inhibition^9^. Dysregulated tissue and circulating miR profiles have been associated with diagnosis, prognosis and sometimes survival in BC^10,11^. Moreover, many dysregulated miRs are reproducibly found in body fluids such as plasma and serum. They are believed to be protected from degradation by association with secreted membrane vesicles or RNA-binding proteins^12^. miRs may represent valuable markers for BC early diagnosis, prognosis as well as conceivable treatment targets^13^. Hence, circulating miRs have the potential to be suitable as minimally invasive biomarkers for early cancer detection. Numerous reports on miRs for BC detection have been published^10^; however, to our knowledge, none were analysed in a BC screening context.

The main aims of the present study were: to identify miR ratios associated with BC through sequencing of small RNAs in a nested case–control study within a large cohort of women attending the BC screening program; to investigate the consistency of the results using Quantitative Reverse Transcription Polymerase Chain Reaction (RT-qPCR), a widespread and low-cost technology; to identify an RT-qPCR based miR ratio signature to be validated in further cohorts of women attending BC screening programs.

## Methods

This case–control study followed the strengthening the reporting of observational studies in epidemiology (STROBE) guidelines for reporting observational studies^14^.

### Study population

ANDROMEDA was a multicentre prospective cohort study on women attending BC screening in two centres in Italy^15^. The eligible population of the study consisted of women of age 46–67 invited to breast screening in the cities of Turin and Biella (two Northern Italian cities in Piedmont), where BC screening is a long-standing practice well known by the people living in the area^16^. Enrolment started in July 2015 for Turin and in May 2016 for Biella, and by the end of the recruitment phase (March 2018), 26,640 women had been included in the study. The cohort has been followed to date through the screening archives to observe the onset of new BC cases. At the time of BC screening appointment, all eligible women were offered to participate. After a detailed explanation of the study protocols, written informed consent was obtained from each participant. Women who agreed to participate in ANDROMEDA were asked, immediately at the enrolment desk, to fill in a short risk questionnaire to collect information on general BC risk factors (reproductive and BC family history, previous breast biopsies, basic physical activity level, body mass index (BMI) and alcohol consumption). In addition, they were asked to fill in a detailed risk questionnaire on diet, physical activity, smoking habits, general state of health and psychological distresses. Life-style information was gathered and employed to build a comprehensive life-style score, as proposed by Romaguera and colleagues^17^ on the EPIC cohort, based on adherence to the World Cancer Research Fund (WCRF) recommendations^18^. The score, ranging from 0 to 7, includes BMI, physical activity, high energy–density foods, plant foods, animal foods, alcoholic drinks, and breastfeeding. The five-year absolute risk was obtained on all samples as estimated by Petracci and colleagues^19^.

Women were also invited to undergo anthropometric measurements (height, weight, body composition, and waist circumference) and to provide a blood sample for serum, plasma and buffy coat storing. Blood specimens were aliquoted, processed and stored at −80 °C.

Incident BC cases were identified through record linkage with screening archives, cancer registries and hospital discharge cards. Intrinsic subtypes of BC were defined using the clinicopathologic surrogate definition reported at the 13th St Gallen International BC Conference^20^. Ethical approval was obtained from the Ethics Committee of each participating center (Ethical and deontological institutional review board of the A.O.U Città della Salute e della Scienza of Turin with the protocol number 78326 on 11.07.2013—and Ethical Committee of Novara with the protocol number 248/CE and study number CE 27/15). The research was performed in accordance with the Declaration of Helsinki guidelines. Informed consent was obtained from all participants. The study was registered in ClinicalTrials.gov with the number NCT02618538, on November 27th, 2015.

### Breast density evaluation

Standard digital mammographies (DM) were performed and read by two expert radiologists. Breast density was calculated during breast examination through two different algorithms: Breast Imaging Reporting and Data System (BI-RADS)^21^ and Tabar^22^. The BI-RADS classified the breast density into category 1—almost fatty (< 25% glandular component); category 2—scattered fibroglandular densities (25–50% glandular); category 3—heterogeneously dense (51–75% glandular); and category 4—extremely dense (> 75% glandular)^21^. Similarly, Tabar classification was adopted as follows: I (balanced proportion of all components of breast tissue with a slight predominance of fibrous tissue), II (predominance of fat tissue), III (predominance of fat tissue with retroareolar residual fibrous tissue), IV (predominantly nodular densities), V (predominantly fibrous tissue)^22^. For subsequent analyses, considering sample distribution and risk classification, the patterns of higher density for both classifications were grouped in a unique category (i.e. BI-RADS 4–5, and Tabar IV–V).

### Selection of cases and controls

**Figure 1:**
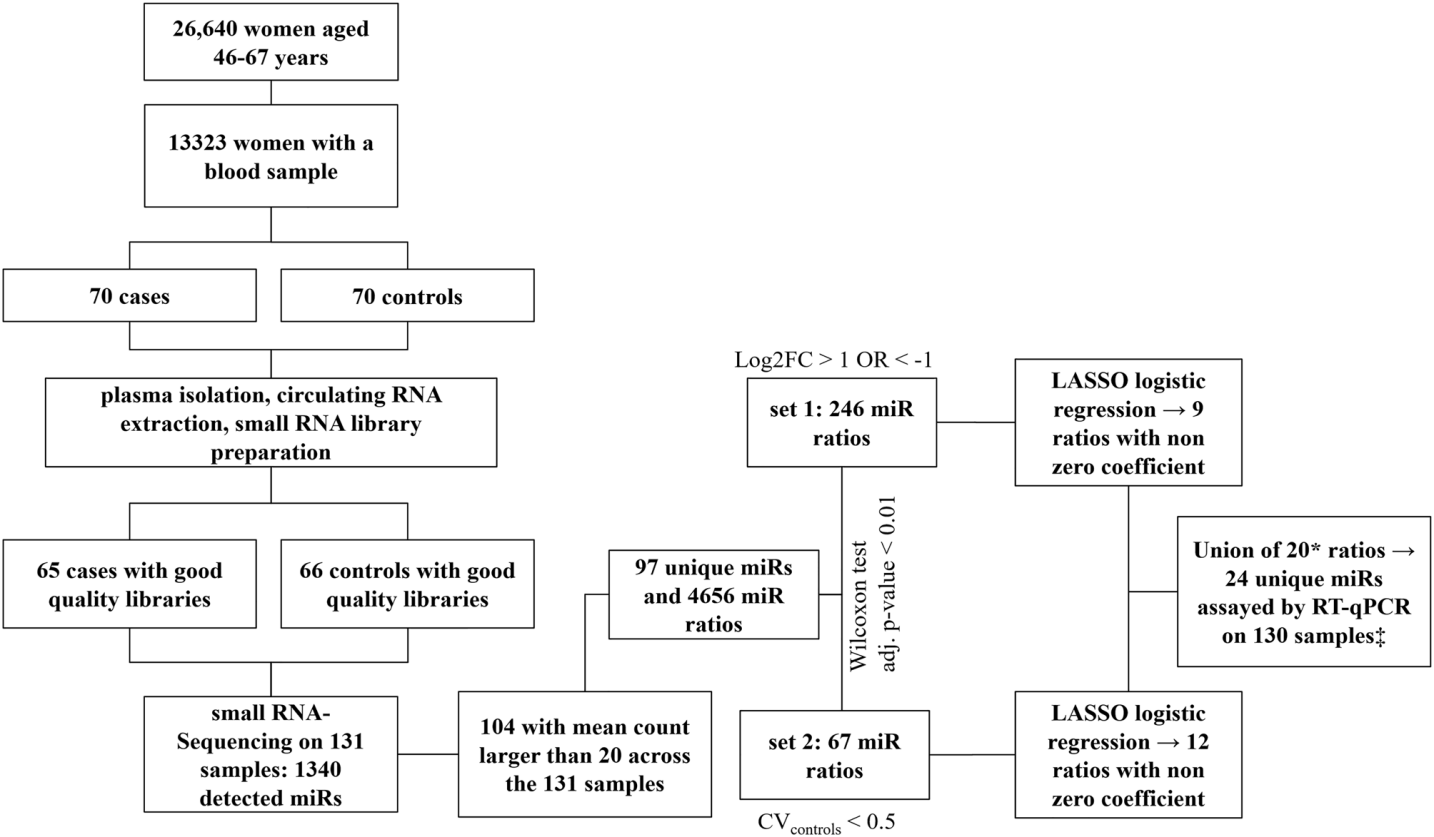
Flow-chart of the study. * The let-7f-5p-2_miR-103a-3p-2 ratio was removed as miR-103a-3p-2 and miR-103a-3p-1, found in the let-7f-5p-1_miR-103a-3p-1 ratio, had almost identical counts and their ratio partners had identical mature miR sequences. ‡ One sample had to be excluded in the RT-qPCR step due to insufficient plasma volume.

For the present study, a case–control study nested within the cohort was conducted. For the nested case–control study, both cases and controls were selected among the participants in the ANDROMEDA cohort study who accepted to provide blood samples at recruitment (n = 14,323, 53.8% of the total). Women with a personal history of BC, with a severe disease or who were unable to give informed consent were excluded from the study. Cases were restricted to women with incident BCs diagnosed within June, 2018 for whom blood was collected before any treatment (n = 70). Moreover, due to the relatively short period of time between blood storage and cases/controls extraction, random sampling, without variable matching, of 70 controls from women who did not experience any BC event before June 2018 was performed. No interval cancers were observed among the controls. The study flow-chart is reported in Fig. 1.

### SNP genotyping and polygenic risk score calculation

For SNP genotyping, genomic DNA was isolated from 200 µl of buffy coat by means of MagMAX DNA Multi-sample Ultra 2.0 kit (Thermo Fisher Scientific, Waltham, MA, USA). DNA concentration and purity were checked by Nanodrop Spectrophotometer (Thermo Fisher). Libraries were prepared starting from 15 ng of DNA and according to the Ion AmpliSeq Library Kit 2.0 protocol for sequencing on the Ion PGM system. The custom panel (Ion AmpliSeq Custom Panel) that selectively covered 80 SNPs target sequences was designed through AmpliSeq Designer (www.ampliseq.com). Ion Xpress Barcodes kit (1–16, 17–32 and 33–48), Ion Ampliseq custom Primer Pool and Ion AmpliSeq Library Kit 2.0-384LV were used in conjunction to obtain libraries. Ion Library Equalizer kit was used to normalise for DNA concentration. Equalised barcoded libraries were pooled and sequenced using Ion PGM Hi-Q OT2 kit and Ion PGM Hi-Q Sequencing Kit on Ion PGM 318 chip V2 on an Ion Torrent PGM (Thermo Fisher Scientific).

Variant calling was performed utilising the Variant Caller plugin within the Torrent Suite Software version 5.10 (Thermo Fisher). The polygenic risk score (PRS) was calculated by adding the multiplications of log odds ratio of each of the 77 SNPs^23^ by the genotype at respective loci (0 for wildtype, 1 for heterozygous variant and 2 for homozygous variant).

### Plasma sample collection and small-RNA sequencing analysis

Plasma isolation from EDTA-tube blood samples, hemolysis check and circulating RNA extraction were carried out as previously described^24^. For library preparation, the Ion Total RNA-Seq kit v2 protocol (Thermo Fisher) with the recommendations for low input RNA quantity was followed as described in^25^. Barcoded primers from Ion Xpress™ RNA-Seq Barcode 01–16 Kit, Thermo Fisher, or synthesised by Eurofins Genomics as custom oligonucleotides (barcodes 17–24) were used. Differentially barcoded small-RNA libraries were pooled and checked by Bioanalyzer System and DNA 1000 Kit (Agilent Technologies) to determine the library dilution required for template preparation. Ion Chef™ System (Thermo Fisher) was used for automated templated Ion Sphere Particles preparation and chip loading. Ion 540 chips (Thermo Fisher) were sequenced using Ion GeneStudio S5 Plus System (Thermo Fisher).

Raw sequence reads were processed using the small-RNA plugin available within the Torrent Suite Software version 5.10 (Thermo Fisher). The reads were aligned to mature miRs using the bowtie2 alignment software^26^, bundled with the plugin. Unmapped reads were further aligned to the whole‑genome to rescue miRbase unaligned reads and count other RNA molecules (tRNAs, rRNAs, mRNAs). miR raw counts were generated using the featureCounts^27^ software from the Subread package 1.5.3. miRs with mean raw counts larger than 20 were selected for further analyses.

To retrieve differentially expressed miR ratios, ratios between the raw counts of all filtered miRs were computed. For any miR pairs with identical count profiles (either due to being clustered or to one miR having two different names), one was removed and a unique identifier was assigned to represent the two miRs. Some miRs with the same name but different chromosomal origin showed different count profiles and were therefore considered as separate. Nevertheless, because the mature sequences of the same miR originating from different genomic loci are the same, in the RT-qPCR validation such occurrences were considered as one single mature miR.

### RT-qPCR validation

The expression of selected miRs was evaluated by RT-qPCR on a CFX-96 machine (Bio-Rad) with TaqMan probes. Four µL of RNA from each sample were reverse transcribed using TaqMan MicroRNA Reverse Transcription kit (Thermo Fisher), with a custom pool of selected miR primers (Thermo Fisher; miR assay IDs: 00546, 002304, 000408, 000390, 002277, 002245, 000468, 000391, 000377, 000382, 000398, 000396, 000407, 001090, 002253, 000420, 002619, 000389, 000580, 000397, 002248, 000442, 000439, 000399). Then 2.5 µL of reverse transcription reaction product were pre-amplified with TaqMan PreAmp Master Mix (Thermo Fisher) and a second pool of selected miR primers (Thermo Fisher). Preamplified samples were diluted with TE buffer and stored at −20 °C for up to one week. A volume of 0.10 µL of diluted preamplified sample was mixed with PCR Master Mix (Thermo Fisher) and water and then transferred in a well of the miR plate. Custom 96 well plates (Thermo Fisher) with 24 miR assays spotted in triplicate were used, allowing for the analysis of one sample per plate.

From the obtained triplicates the mean Cycle threshold (Ct) was calculated. Non-detects were replaced with the Ct value of 40. If a replicate within the triplicate was one standard deviation away from the mean it was excluded and a new Ct mean was calculated. In order to calculate the ratio between miR X and miR Y, the following equation was used as explained by Deng and colleagues in 2019^28^: Ct_mean_(Y) − Ct_mean_(X).

### Statistical analysis

On small-RNA sequencing data, Mann–Whitney U test was performed to compare miR ratios between cases and controls and p-values were corrected using the Benjamini–Hochberg method. The fold change was calculated based on the median in cases and controls. Significantly different ratios between cases and controls (Mann–Whitney U test adjusted *p*-*value* ≤ 0.01) with a fold change > 2 or < 0.5, were selected as strategy 1, while ratios with a coefficient of variation < 0.5 within controls and significantly different between cases and controls, without setting any criteria on fold change, were selected as strategy 2. The ratios from the two strategies were further analysed by a Least Absolute Shrinkage and Selection Operator (LASSO) logistic regression^29^. Five-fold cross-validation was used to preliminarily assess the performance of the model-selected ratios, separately for the two strategies defined above. Thus, the sample was randomly divided into five groups, called folds, and the LASSO logistic model was trained on five minus one folds. Then, the performances of the resulting model were evaluated on the remaining part of the data. This procedure was repeated for each fold and the performances obtained each time averaged. The following performance measures were considered: calibration intercept, Cox’s measure of spread (often called “calibration slope”)^30^, scaled Brier score, and area under the receiver operating characteristic curve (AUC). The first three measures mainly relate to the agreement between the observed outcomes and the outcomes predicted from the model. For the intercept and scaled Brier, ideal values should be as close to zero as possible, whereas for the Cox calibration slope close to one. The AUC refers to the model's ability to discriminate between individuals with a different outcome and the ideal values should be close to one. Cross-validation was performed mainly to select promising ratios to validate by RT-qPCR.

Using standard logistic regression, univariate odds ratios (OR) and corresponding 95% confidence intervals (CIs) were obtained on RT-qPCR data. The linearity assumption between a continuous predictor and the logit of risk was inspected through the Locally Weighted Scatterplot Smoothing (LOWESS) and restricted cubic splines, whereas for ordinal variables the Cochran-Armitage trend test was used to assess the presence of a linear trend. To derive a ratio-based signature as well as to preliminarily investigate the potential added value of miR ratios over more conventional BC risk factors and their potential independent role in predicting BC risk, the LASSO logistic regression was used. Three models were then fitted: one using miR ratios only, one combining the ratios with other potential BC risk factors and one on BC risk factors alone. To select BC-associated factors for inclusion in the model together with the miR ratios, we assessed the association between BC detection and other factors such as PRS, demographic, family, reproductive and screening history, life-style, and breast density information, as well as any interaction between them relevant to BC. The discriminatory ability of the models was assessed using AUC (with reported 95% CIs), whereas the Youden index was used as the criterion to derive a cut-off point on the predicted probabilities and compute sensitivity and specificity. The paired Delong test was used to compare the discrimination among different models.

The association between demographic, life-style, anthropometric and reproductive factors as well as cancer characteristics and the selected ratios were performed using the Mann–Whitney U test or the Kruskal Wallis test, as appropriate, for categorical covariates and using the Spearman correlation coefficient for continuous covariates. Continuous variables were reported by mean ± standard deviation (sd) or median and I and III quartiles, as appropriate, whereas categorical variables were reported as natural frequency and percentage. All analyses were performed using the open-source statistical computing environment R^31^. The main packages used were: glmnet^32^ for LASSO, ROCR^33^ and OptimalCutpoints^34^ for plotting ROC curves and cut-off search.

### Target enrichment analysis

Target and functional enrichment analyses were performed on the miRs making up the ratio signature associated with BC detection using the Mienturnet online software^35^. In all the mentioned analyses the miRTarBase database was used as it includes experimentally validated miRs.

## Results

### Population characteristics

After RNA extraction from the plasma of 70 cases and 70 controls and library preparation, nine samples were excluded due to poor quality. Thus, the final cohort consisted of 65 cases and 66 controls (Fig. 1). The general characteristics of the study population are reported in Table 1. The only variables that showed a significant association with BC detection in this cohort were: BMI, breast density and WCRF score. The characteristics of cases are reported in Table 2, separately for invasive and in situ tumours. Cases were diagnosed on average 3 ± 2 months after blood collection. Fifty-five women were diagnosed with invasive breast tumours and eight with in situ lesions. The most frequent histotype was ductal (56.0% of invasive and 37.5% of in situ BCs) and the majority of cancers were stage IA (87.5%), Her2 negative (86.5%) and Ki-67 negative (76.5%). Further, DNA isolated from buffy coat was used to calculate the 77 SNP PRS, which was not found to be significantly associated with BC in our cohort (Table 1).

**Table 1:** Demographic, family, reproductive and screening history, life-style, anthropometric measurements, education, breast density, PRS and 5-year absolute BC risk information is reported. Results of univariate logistic regression for each variable are also reported.

	Controls (*n* = 66)		Cases (*n* = 65)		OR (95% CI)	*P*
	*n*	(%)	*n*	(%)		
Age at enrollment, years						
Mean ± sd	57.82 ± 5.92		59.15 ± 6.00		1.04 (0.98–1.10)	0.201
Centre						
Biella	20	(30.30)	16	(24.62)	1 (ref)	
Torino	46	(69.70)	49	(75.38)	1.33 (0.62–2.91)	0.467
Previous negative second-level screening rounds						
0	62	(93.94)	59	(90.77)	1 (ref)	
≥ 1	4	(6.06)	6	(9.23)	1.58 (0.43–6.43)	0.497
Previous benign biopsies						
0	57	(86.36)	49	(77.78)	1 (ref)	
≥ 1	9	(13.64)	14	(22.22)	1.81 (0.73–4.69)	0.207
Missing			2			
Education						
Low	21	(32.31)	22	(34.38)	1 (ref)	
Medium	31	(47.69)	28	(43.75)	0.86 (0.39–1.90)	0.712
High	13	(20.00)	14	(21.88)	1.03 (0.39–2.71)	0.955
Missing	1		1			
Nr. of first-degree relatives with BC						
0	58	(87.88)	56	(88.89)	1 (ref)	
≥ 1	8	(12.12)	5	(7.94)	0.65 (0.19–2.06)	0.469
Missing			2			
Age at menarche, years						
≤ 11	22	(33.33)	19	(29.69)	1 (ref)	
12–13	33	(50.00)	33	(51.56)	1.16 (0.53–2.54)	0.713
≥ 14	11	(16.67)	12	(18.75)	1.26 (0.45–3.55)	0.654
Missing			1			
Age at first full pregnancy, years						
Nulliparous	19	(28.79)	11	(16.92)	0.39 (0.13 –1.13)	0.087
≤ 19	3	(4.55)	1	(1.54)	0.22 (0.01–2.02)	0.219
20–24	11	(16.67)	15	(23.08)	1 (ref)	
25–29	21	(31.82)	16	(24.62)	0.51 (0.18–1.41)	0.198
≥ 30	12	(18.18)	22	(33.85)	1.39 (0.47–4.13)	0.545
Contraceptive therapy						
No OR use < 1 year	29	(45.31)	31	(48.44)	1 (ref)	
1–4 years	10	(15.62)	5	(7.81)	0.47 (0.13–1.48)	0.210
≥ 5 years	25	(39.06)	28	(43.75)	1.04 (0.50–2.20)	0.902
Missing	2		1			
Breastfeeding						
Nulliparous OR no breastf. OR breastf. < 6 months	42	(63.64)	39	(60.94)	1 (ref)	
≥ 6 months	24	(36.36)	25	(39.06)	1.12 (0.55–2.29)	0.751
Missing	–		1			
Menopausal status						
Not in menopause	12	(18.18)	12	(18.75)	1 (ref)	
Menopause	54	(81.82)	52	(81.25)	0.74 (0.30–1.78)	0.504
Missing	–		1			
HRT use						
Not in menopause	12	(18.18)	12	(18.75)	1 (ref)	
No HRT use OR HRT use < 1 year	43	(65.15)	45	(70.31)	1.04 (0.42–2.60)	0.921
≥ 1 year	11	(16.17)	7	(10.94)	0.63 (0.18–2.18)	0.475
Missing	–		1			
Measured BMI, kg/m^2^						
Mean ± sd	25.76 ± 5.15		28.02 ± 6.24		1.07 (1.01–1.15)	0.029
Waist circumference, cm						
Mean ± sd	88.00 ± 11.83		92.69 ± 17.55		1.02 (1.00–1.05)	0.087
Missing	2		2			
Level of occupational physical activity at age 30–39 years						
Exclusively/maily sitting	17	(25.80)	23	(35.90)	1 (ref)	
Standing or average	43	(65.20)	34	(53.10)	0.58 (0.27–1.26)	0.172
Heavy or very heavy	6	(9.10)	7	(10.90)	0.86 (0.24–3.12)	0.817
Missing	–		1			
Level of leisure time physical activity at 30–39 years						
< 2 h/week	35	(53.00)	34	(53.10)	1 (ref)	
≥ 2 h/week	31	(47.00)	30	(46.90)	1.00 (0.50—1.99)	0.991
Missing	1		1			
Alcohol habit						
Never drinker or ex drinker	16	(24.24)	18	(29.69)	1 (ref)	
Drinker, also occasionally	50	(75.76)	45	(70.31)	0.76 (0.35–1.65)	0.485
Missing	–		1			
Smoking habit						
Never smoker	31	(47.69)	26	(41.94)	1 (ref)	
Ex-smoker	19	(29.23)	25	(40.32)	1.57 (0.71–3.50)	0.265
Occasionally/Smoker	15	(23.08)	11	(17.74)	0.87 (0.34–2.22)	0.779
Missing	1		3			
BI-RADS breast density						
1	21	(31.82)	21	(32.31)	1 (ref)	
2	34	(51.52)	27	(41.54)	0.79 (0.36–1.75)	0.566
3 or 4	11	(16.67)	17	(26.15)	1.55 (0.59–4.15)	0.379
TABAR breast density						
1	22	(33.33)	10	(15.38)	1 (ref)	
2	25	(37.88)	23	(35.38)	2.02 (0.80–5.32)	0.141
3	6	(9.09)	7	(10.77)	2.57 (0.69–10.01)	0.162
4 or 5	13	(19.70)	25	(38.46)	4.23 (1.59–11.97)	0.005
5-year absolute breast cancer risk estimate						
Mean ± sd	0.017 ± 0.008		0.020 ± 0.012		1.49 (0.71–3.23)	0.296
Missing	2		4			
WCRF/AICR score						
Mean ± sd	5.52 ± 0.98		5.12 ± 1.11		0.68 (0.47–0.96)	0.034
PRS						
Mean ± sd	0.98 ± 0.43		1.00 ± 0.41		1.09 (0.47–2.51)	0.842
Missing	4		–			

*OR* odds ratio, *CI* confidence interval, *sd* standard deviation, *HRT* hormone replacement therapy, *BI-RADS* breast imaging reporting and database system: 1 almost fatty; 2 scattered fibroglandular densities; 3 heterogeneously dense; 4 extremely dense.

*TABAR* Tabàr’s breast density classification system; as I (balanced proportion of all components of breast tissue), II (predominance of fat tissue), III (predominance of fat tissue with retroareolar residual fibrous tissue), IV (predominantly nodular densities), V (predominantly fibrous tissue).

*WCRF/AICR* world cancer research fund/American institute for cancer research.

Education categories: Low (Secondary school or lower), Medium (High school/technical school), High (bachelor’s degree or higher).

*PRS* polygenic risk score.

**Table 2:** Histological and molecular subtype characteristics of invasive and in situ breast cancer cases (n = 65).

Invasive (n = 57)			In situ (n = 8)		
	*n*	(%)		*n*	(%)
Histotype			Histotype		
Ductal NOS	30	56.0	Ductal NOS	3	37.5
Lobular	8	18.0	Solid	1	12.5
Tubular	4	8.0	Micropapillary	1	12.5
Other	9	18.0	Papillary	1	12.5
Missing	6		Other	2	25.0
Grade			Grade		
I	18	34.7	I	2	25.0
II	25	51.0	II	2	25.0
III	6	14.3	III	4	50.0
Missing	8		Tumour size [I27], mm		
pT			1–10	4	50.0
1a-1b-1mic	25	48.1	11–20	2	25.0
1c	24	42.6	21 +	2	25.0
2 +	5	9.3			
Missing	3				
Tumour size, mm					
1–10	25	46.3			
11–20	24	44.4			
21 +	5	9.3			
Misssing	3				
Stage					
IA	42	87.5			
IIA	3	6.3			
IIIC	2	4.2			
IV	1	2.1			
Missing	9				
Molecular subtypes					
ER					
Negative	8	15.1			
Positive (> 10%)	45	84.9			
Missing or undetermined	4				
PgR					
Negative	17	32.1			
Positive (> 10%)	36	69.9			
Missing or undetermined	4				
Her2					
Negative	45	86.5			
Positive	7	13.5			
Missing or undetermined	5				
Ki-67					
Negative	39	76.5			
Positive (> 20%)	12	23.5			
Missing or undetermined	6				
Intrinsic subtype					
Luminal A-like	27	52.9			
Luminal B-like (HER2 negative)	13	25.5			
Luminal B-like (HER2 positive)	5	9.8			
HER2 positive (non luminal)	2	3.9			
Triple negative	4	7.8			
Missing	6				

*NOS* not otherwise specified, *pT* pathologic evaluation of tumour size, *ER* estrogen receptor, *PgR* progesterone receptor, *Her2* human epidermal growth factor receptor 2.

### Identification of circulating miR ratios through small-RNA sequencing

Out of 1340 circulating miRs detected by small-RNA sequencing, 104 had a mean count larger than 20 across the 131 samples, resulting in 97 unique miRs and 4656 miR ratios (Fig. 1). Based on the Mann–Whitney U test, 886 ratios were differentially expressed between cases and controls in the first set and 67 in the second. miR ratios with less than twofold modulation were removed from the first set, leaving 246 ratios. Fitting a LASSO logistic model separately on each set of ratios obtained from the two strategies, resulted in 9 and 12 ratios associated with BC risk (Fig. 1 and Table S.1 in Additional file 1), respectively. The AUCs of the selected ratios, computed on the original sample, are reported in Table 3 and ranged from 0.66 to 0.81. The overall performance of the two LASSO models as assessed by five-fold cross-validation, is also reported in Table 3. The calibration intercept, which is an assessment of calibration-in-the-large, had a target value of 0, whereas the Cox slope slightly deviated from its target value of 1. In particular, for strategy 1, the model including the 9 ratios tended to produce risk estimates that were too moderate whereas, for strategy 2, the model including the 12 ratios produced estimates that were too extreme, that is, that were too high for women at high risk and too low for women at low risk. For both strategies, the Brier score suggested an absence of disagreement between the observed outcome and the prediction. Discrimination, as measured by the AUC, was 0.80 and 0.79 for the first and second strategy, respectively.

**Table 3:** Performance of the selected miR ratios on small-RNA sequencing data.

	Strategy 1	Strategy 2
AUC in the original sample		
miR-335-5p_let-7f.-5p-2	0.76	
miR-199a-3p-2_let-7a-5p-2	0.71	
miR-199a-3p-2_let-7f.-5p-2	0.72	
let-7a-5p-2_miR-22-3p	0.81	
let-7a-5p-2_miR-320a	0.80	
let-7f.-5p-1_miR-19b-3p-1	0.79	
miR-27a-3p_miR-122-5p	0.71	
let-7f.-5p-2_miR-146a-5p	0.78	
miR-15b-5p_miR-16-5p-1	0.71	
miR-26b-5p_miR-142-5p		0.68
let-7a-5p-2_miR-106b-5p		0.77
let-7f.-5p-1_miR-103a-1		0.76
let-7f.-5p-2_miR-103a-2		0.74
miR-93-5p_miR-19b-3p-1		0.69
miR-22-3p_miR-19b-3p-2		0.67
miR-101-3p-2_miR-19b-3p-1		0.68
miR-30d-5p_miR-20a-5p		0.68
let-7b-5p_miR-19b-3p-1		0.74
miR-15a-5p_miR-16-5p-2		0.66
miR-20a-5p_miR-19b-3p-1		0.77
miR-21-5p_miR-23a-3p		0.68
Performance of the strategies by fivefold cross-validation		
Calibration intercept	0.051	0.024
Calibration slope	1.269	0.840
Scaled brier	0.060	0.060
AUC	0.797	0.791

AUC: area under the ROC curve.

### Cross-platform validation

By combining strategy 1 and strategy 2, a total of 20 ratios, which included 24 unique miRs, were further analysed by RT-qPCR on 130 samples (Fig. 1). One ratio (let-7f-5p-2_miR-103a-3p-2) was removed as miR-103a-3p-2 and miR-103a-3p-1, found in the let-7f-5p-1_miR-103a-3p-1 ratio, had identical counts in all but two samples (with negligible difference) and their ratio partners had identical mature miR sequences. In addition, one control sample had to be excluded from the RT-qPCR step due to insufficient plasma volume. Based on the median ratio values, small-RNA sequencing and RT-qPCR yielded overall concordant values in cases and controls. However, four ratios showed an opposite trend (Fig. S.1 in Additional file 2—comparing corresponding miR ratios between the two platforms). Seven ratios had a significantly positive Spearman rank correlation coefficient (*p-value* < 0.01) between the two platforms (miR-26b-5p_miR-142-5p, miR-101-3p_miR-19b-3p, let-7b-5p_miR-19b-3p, let-7f-5p_miR-19b-3p, let-7a-5p_miR-320a, miR-27a-3p_miR-122-5p, miR-199a-3p_let-7a-5p), with the coefficients ranging from 0.23 to 0.34 (Table S.2 in Additional file 1). Albeit not significantly correlated, 9 ratios had positive correlation coefficients < 0.20 and four had negative coefficients between the compared platforms. AUCs for each ratio as well as the univariable logistic regression results are reported in Table 4. Overall, the individual accuracy ranged from 0.48 to 0.65 and three ratios were associated with BC at a nominal 5% level of significance: miR-26b-5p_miR-142-5p, let-7a_miR-22-3p, and miR-199a-3p_let-7a-5p.

**Table 4:** Results from standard univariable logistic regression and AUCs on RT-qPCR data.

miR ratio	Expression in cases		Expression in controls		OR	(95% CI)	*P*	AUC
	Median	[IQ range]	Median	[IQ range]				
miR-26b_miR-142-5p	5.84	[5.08–6.08]	6.05	[5.61–6.44]	0.48	(0.28–0.77)	0.005	0.65
let-7a_miR-22-3p	5.48	[3.54–7.81]	6.94	[4.08–9.89]	0.85	(0.73–0.98)	0.026	0.63
miR-199a-3p_let-7a-5p	1.66	[1.08–2.21]	1.24	[0.92–1.90]	1.64	(1.05–2.63)	0.033	0.61
miR-93-5p_miR-19b-3p	− 3.07	[− 3.45 to − 2.79]	− 3.29	[− 3.52 to − 3.04]	2.05	(1.00–4.50)	0.059	0.61
miR-199a-3p_let-7f.-5p	3.65	[2.97–4.15]	3.20	[2.74–3.93]	1.41	(0.97–2.08)	0.077	0.58
miR-15a-5p_miR-16-5p	− 19.10	[**− **20.43 to − 16.51]	− 17.41	[− 18.50 to − 16.96]	0.82	(0.65–1.02)	0.087	0.63
miR-22-3p_miR-19b-3p	− 11.04	[− 13.85 to − 9.74]	− 12.53	[− 15.49 to − 10.03]	1.12	(0.98–1.30)	0.113	0.60
let-7b-5p_miR-19b-3p	− 3.19	[− 3.61 to − 2.69]	− 2.83	[− 3.38 to − 2.54]	0.74	(0.47–1.13)	0.176	0.59
let-7f.-5p_miR-146a-5p	− 7.82	[− 8.60 to − 7.42]	− 7.57	[− 8.41 to − 6.71]	0.83	(0.62–1.10)	0.210	0.59
miR-27a-3p_miR-122-5p	0.78	[− 0.75–2.06]	0.65	[− 0.80–1.48]	1.12	(0.93–1.36)	0.241	0.54
let-7a-5p_miR-106b-5p	− 0.17	[− 0.68–0.38]	0.07	[− 0.36–0.70]	0.78	(0.50–1.20)	0.270	0.59
miR-15b-5p_miR-16-5p	− 10.13	[− 10.92 to − 8.80]	− 10.12	[− 10.88 to − 9.32]	1.12	(0.92–1.38)	0.276	0.52
miR-335-5p_let-7f.-5p	1.44	[0.78–2.27]	1.03	[0.46–2.03]	1.18	(0.86–1.64)	0.319	0.58
miR-20a-5p_miR-19b-3p	− 0.50	[− 0.69 to − 0.14]	− 0.48	[− 0.76 to − 0.21]	0.78	(0.45–1.28)	0.351	0.48
let-7f.-5p_miR-19b-3p	− 7.79	[− 8.36 to − 7.08]	− 7.49	[− 8.16 to − 6.66]	0.87	(0.64–1.16)	0.355	0.57
let-7f.-5p_miR-103a-3p	− 1.23	[− 1.88 to − 0.79]	− 1.19	[− 1.66 to − 0.82]	0.86	(0.61–1.20)	0.390	0.53
let-7a-5p_miR-320a	− 4.30	[− 4.94 to − 3.49]	− 4.08	[− 4.63 to − 3.16]	0.88	(0.65–1.17)	0.399	0.57
miR-30d-5p_miR-20a-5p	− 4.27	[− 4.79 to − 4.04]	− 4.29	[− 4.62 to − 4.03]	0.97	(0.63–1.50)	0.905	0.50
miR-21-5p_miR-23a-5p	4.34	[3.93–4.91]	4.30	[3.80–4.95]	1.02	(0.66–1.58)	0.917	0.52
miR-101-3p_miR-19b-3p	− 8.09	[− 8.63 to − 7.65]	− 8.17	[− 8.64 to − 7.69]	0.99	(0.70–1.41)	0.969	0.49

*OR* odds ratio, *CI* confidence interval, *AUC* area under the ROC curve.

### Identification of robust miR ratios for BC detection

**Figure 2:**
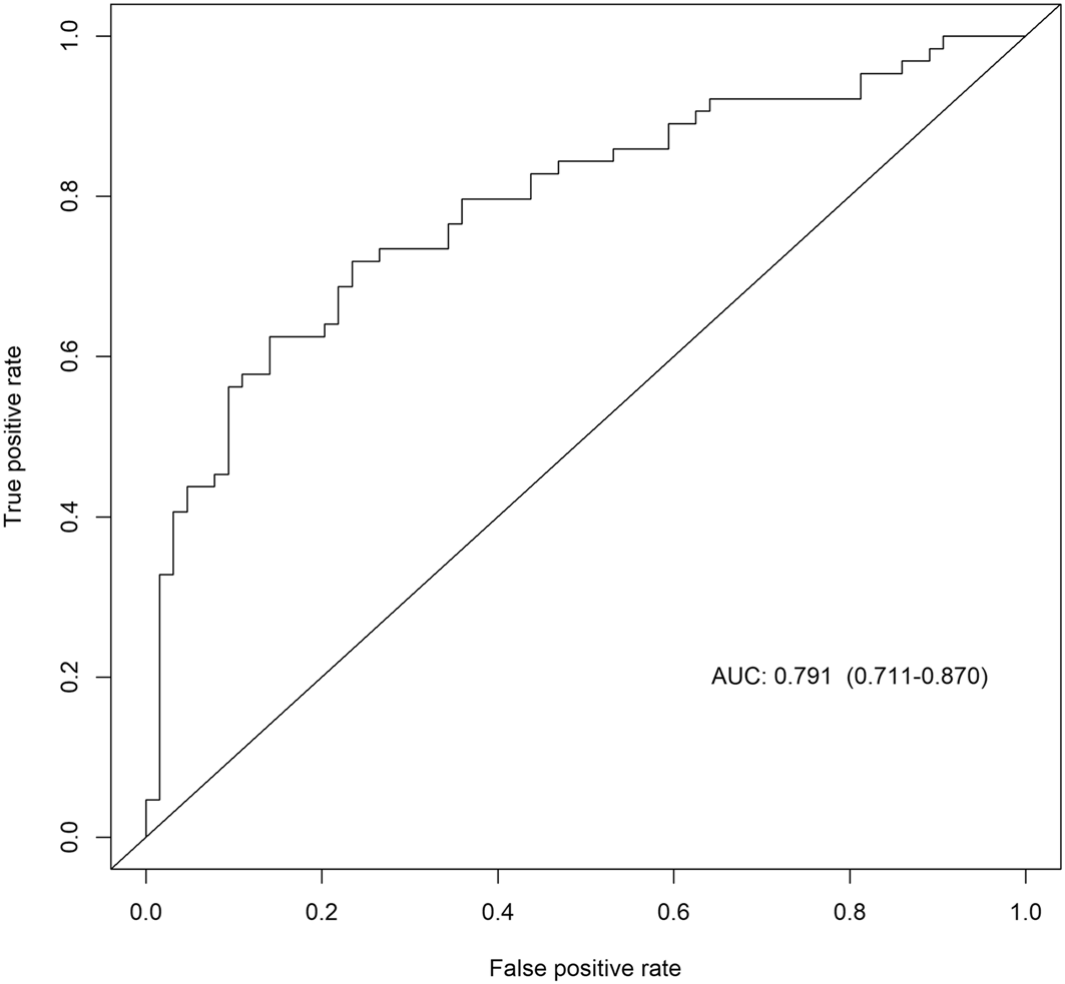
ROC curve of the multivariable model on 7 miR ratios and other non-molecular variables.

To obtain the most promising miR ratios for BC detection and test their added value compared with other variables associated with BC (Table 1), three LASSO logistic regression models were fitted (see “Statistical analysis” section). The first model selected 6 ratios with non-zero coefficients: miR-199a-3p_let-7a-5p, miR-26b-5p_miR-142-5p, let-7b-5p_miR-19b-3p, miR-101-3p_miR-19b-3p, miR-93-5p_miR-19b-3p, let-7a-5p_miR-22-3p (Table 5). The corresponding AUC was 0.73 (95% CI 0.64–0.82); Youden’s optimal cut-off was 0.51 with corresponding sensitivity and specificity of 65.2% and 75.0%, respectively. The second model included the abovementioned ratios as well as miR-21-5p_miR-23a-3p, Tabar’s breast density classification and WCRF scores (as continuous variables), BMI (≥ 30 vs. < 30), menopausal status (yes vs. no) and an interaction term between the last two variables. Menopause and the interaction term were included due to the known different effects of BMI in pre- and post-menopausal women^36^. The AUC associated with this model was 0.79 (95% CI 0.71–0.87); the Youden’s cut-off was 0.50 and the associated sensitivity and specificity were 71.9% and 76.6%, respectively (Fig. 2). For comparison, the model including the abovementioned non-molecular factors only had an associated AUC of 0.72 (95% CI 0.63–0.81); at the optimal cut-off value of 0.51, sensitivity and specificity were 68.8% and 70.3%, respectively. The Delong test on the model with non-molecular variables and the model which additionally included the miR ratios revealed a significant difference between their AUCs (Z = − 2.0857, *p* = 0.03701). The other model comparisons yielded insignificant AUC differences.

**Table 5:** Results from LASSO logistic regression for the risk of BC on RT-qPCR performed on the miR ratio only (Model 1) and also including non-molecular variables (Model 2).

	Model 1	Model 2
	Coefficients	Coefficients
Intercept	1.32	1.14
miR-199a-3p_let.7a-5p	0.16	0.16
miR-26b-5p_miR-142-5p	− 0.16	− 0.13
let-7b-5p_miR-19b-3p	− 0.18	− 0.27
miR-101-3p_miR-19b-3p	− 0.07	− 0.10
miR-93-5p_miR-19b-3p	0.47	0.57
let-7a-5p_miR-22-3p	− 0.04	− 0.05
miR-21-5p_miR-23a-3p		0.02
Menopausal status		− 0.03
BMI		0.36
Menopausal status*BMI^‡^		0.15
WCRF/AICR score		− 0.16
TABAR		0.27

*TABAR* tabàr’s breast density classification system, *WCRF/AICR* world cancer research fund/American institute for cancer research.

^‡^Interaction between menopausal status and BMI.

Five of the seven miR ratios in the final model had significant associations with clinicopathological characteristics based on the RT-qPCR data (Fig. S.2 in Additional S.2). Namely, miR-93-5p_miR-19b-3p was found to be lower in ER+ compared to ER− invasive BC patients (*p* = 0.037). miR-26b-5p_miR-142-5p was lower in ki-67+ compared to ki-67− invasive BCs (*p* = 0.048). Interestingly, miR-21-5p_miR-23a-3p was higher in ER+ than in ER− invasive BC patients (*p* = 0.030), in PgR+ versus PgR− (*p* = 0.036) as well as in ki-67− in contrast to ki-67+ BC invasive patients (*p* = 0.033). Lastly, let-7a-5p_miR-22-3p was lower in ductal compared to other BC histotypes (*p* = 0.050).

### Target enrichment analysis results

Functional target enrichment analysis was performed on miRs making up the ratio signature in the model with non-molecular variables. Due to the software limitation of possible number of miRs in a single functional enrichment analysis, we excluded let-7b-5p as it has a very similar mature sequence and function to let-7a-5p, which was included in the analysis. Functional enrichment on the 10 miRs revealed their general involvement in cancer and breast cancer pathways, PI3K—Akt signaling pathway as well as the ATM-dependent DNA damage response (Wikipathways). Additionally, their targets were involved in androgen receptor signalling and EGF/EGFR signalling pathways (Supplementary Fig. S.3). Messenger RNAs of 12 genes were commonly targeted by at least 5 of the 10 analysed miRs, with the most targeted genes being the tumour suppressor phosphatase and tensin homolog (*PTEN)* (7 miRs) followed by Nuclear FMR1 Interacting Protein 2 (*NUFIP2)* (6 miRs).

## Discussion

Mammography is currently the gold standard examination for BC screening, and organised population screening programs have significantly reduced BC mortality. However, BC screening has known limitations (i.e. false positives, overdiagnosis and interval cancers) that could be overcome with accurate non-invasive markers capable of complementing or tailoring mammographic screening^37^. Although they have been proposed for many different cancers, including BC, in diagnostic/prognostic contexts, to our knowledge, no studies focused on the potential role of circulating miRs in asymptomatic women undergoing general mammographic screening. In this study, we aimed to identify new circulating biomarkers associated with BC in plasma samples from a nested case–control study within a large cohort of women attending BC screening^15^. Importantly, BC status of cases and controls was not known before blood sampling as blood collection occurred before obtaining the first-level mammography result and none of the participants experienced any symptoms which might indicate BC. Small-RNA sequencing was first applied and, to facilitate subsequent validation by RT-qPCR (where no suitable normalisers are available) as well as cross-platform comparison, miR ratios in place of the individual miRs were considered throughout the analyses. A total of 20 ratios, made up of 24 unique miRs, were obtained as potential biomarker candidates based on the small-RNA sequencing data. The 24 miRs were then further tested with RT-qPCR on the same initial cohort. To assess the diagnostic ability of the candidate miR biomarkers and make a comparison to other non-molecular variables associated with BC in our cohort, three diagnostic models were built: a model on non-molecular variables only, a model on miR ratios only and a model with miR ratios and non-molecular variables combined. In the multivariable model which included five non-molecular variables, a signature of 7 miR ratios consisting of 11 unique miRs was identified. Four of the seven ratios were found to be associated with clinicopathological characteristics among cases, such as the ER or ki-67 status, implying a possibly direct function of the miRs making up the ratios in cancer formation and progression. The target enrichment analysis of the miRs making up the 7 ratios revealed that the genes they target are involved in cancer pathways including BC. Importantly, all 10 analysed miRs were enriched in the PI3-Akt signalling pathway, which is relevant to tumour progression and endocrine resistance in BC^38^. The genes commonly targeted by the majority of the 10 miRs were *PTEN* and *NUFIP2*. *PTEN* is a known tumour suppressor blocking the PI3K signalling^39^, while *NUFIP2* is an RNA-binding protein^40^.

Five of the unique 11 miRs, from the model including miR ratios and non-molecular variables, were previously detected as potential diagnostic circulating biomarkers in other BC studies whose TNM stage distribution of cases also roughly matched the distribution of stages observed in BC screening programs^41^. The 5 miRs are: let-7a-5p^42^, miR-19b-3p^43–45^, let-7b-5p^43,46^, miR-93-5p^43,47^ and miR-21-5p^48^. Barring miR-19b-3p and miR-21-5p, the mentioned miRs are believed to be tumour suppressors or to have a protective role in BC tissue^49–51^. For instance, let-7a is believed to suppress BC cell migration by downregulating the CC chemokine receptor 7^52^. Moreover, through IL-8 regulation, let-7b suppresses the cancer-promoting nature of BC-associated fibroblasts^49^. The diagnostic accuracy of the miR ratios (both alone and when combined with non-molecular variables) showed promising results with (AUCs of 0.73 and 0.79, respectively) which are comparable to what has been obtained in previous studies^44,53–56^. For example, Fang et al. 2019, which also utilised a plasma-based miR ratio model (5-ratios) by cross-platform validation on 131 samples, obtained a sensitivity and specificity of 71.7% and 78.2%, respectively^56^. The 5 ratios were made up of 7 unique miRs, of which none match the miRs in our final model. This might, in part, be due to the different reference populations or variations in experimental and analytical methods. For instance, unlike our study, Fang et al. 2019 did not calculate the pairwise ratios on the small-RNA sequencing data but only on RT-qPCR. Another study performed in 2015^44^, conducted on a profiling (N = 86) and validation cohort (N = 196), reported an 8-miR model (miR-16, let-7d, miR-103, miR-107, miR-148a, let-7i, miR-19b, miR-22-5p) with a 91% sensitivity and 49% specificity and an AUC of 0.81. One of the miRs in their model, miR-19b, was included in three ratios obtained in our final models.

Among the majority of similar studies involving circulating cell-free miRs, controls usually come from healthy donors recruited in a separate setting from the cases, which were generally diagnosed before blood sampling^57^. Hence, the primary strength of our study is that all samples came from the screening setting and were prospectively sampled with the limitation of a relatively small sample size^58^. Additionally, most of the published studies on diagnostic cell-free circulating miRs were based on endogenous or exogenous miR normalisers^10^. However, an essential aspect of standardising the cell-free circulating miR analysis is the normalisation method^10,59^, and utilising miR ratio-based values is a good step forward to overcome the lack of optimal endogenous or exogenous normalisers^28^. Moreover, blood sampling before biopsy and before knowing the BC status might provide a higher chance of obtaining a real un-confounded circulating miR profile.

## Conclusions

We identified candidate miR ratios which could assist, together with non-molecular parameters, in early BC detection in the setting of mammographic screening and can be measured through a widespread and low-cost technique. This is the first study reporting circulating miRs for BC detection in a screening setting. Considering the relatively small number of patients and lack of external validation, further evaluation of the presented miR biomarkers on a larger cohort is warranted.

## Supplementary Information

Supplementary Tables.

Supplementary Figures.

## Data Availability

The small-RNA sequencing data generated in this study, both raw and processed, has been deposited in the NCBI Gene Expression Omnibus public repository and is accessible through GEO Series accession number GSE210329 (https://www.ncbi.nlm.nih.gov/geo/query/acc.cgi?acc=GSE210329). The RT-qPCR data is available upon request from the corresponding author.

## Abbreviations

BC: Breast cancer
SNP: Single nucleotide polymorphism
miR: MicroRNA
RT-qPCR: Quantitative reverse transcription polymerase chain reaction
STROBE: The strengthening the reporting of observational studies in epidemiology
BMI: Body mass index
DM: Digital mammography
WCRF: World cancer research fund
AICR: American institute for cancer research
BI-RADS: Breast imaging reporting and data system
Ct: Cycle threshold
LASSO: Least absolute shrinkage and selection operator
AUC: Area under the ROC curve
OR: Odds ratio
CI: Confidence interval
LOWESS: Locally weighted scatterplot smoothing
sd: Standard deviation
HRT: Hormone replacement therapy
ER: Estrogen receptor
PgR: Progesterone receptor
Her2: Human epidermal growth factor receptor 2
