# Supplementary material for: Plasma microRNA ratios associated with breast cancer detection in a nested case–control study from a mammography screening cohort
Source: Sci Rep. 2023 Jul 25;13:12040. doi: 10.1038/s41598-023-38886-0 (PMC10368693; doi:10.1038/s41598-023-38886-0)
Supplement: Supplementary file 1 — Supplementary Tables. [file 41598_2023_38886_MOESM1_ESM.docx]

Table S.1 Results from LASSO logistic regression for risk of BC for the two strategies on RNA-seq data

| Strategy 1 (*n*_miR-ratio_ set 1=246) | coefficients | Strategy 2 (*n*_miR-ratio_ set 2=67) | coefficients |
| --- | --- | --- | --- |
| intercept | 1.12 | intercept | 2.95 |
| miR-335-5p_let-7f-5p-2 | 0.002 | miR-26b-5p_miR-142-5p | -0.13 |
| miR-199a-3p-2_let-7a-5p-2 | 0.26 | let-7a-5p-2_miR-106b-5p | -0.82 |
| miR-199a-3p-2_let-7f-5p-2 | 0.0005 | let-7f-5p-1_miR-103a-1 | -1.11 |
| let-7a-5p-2_miR-22-3p | -0.54 | let-7f-5p-2_miR-103a-2 | -0.65 |
| let-7a-5p-2_miR-320a | -0.37 | miR-93-5p_miR-19b-3p-1 | -2.80 |
| let-7f-5p-1_miR-19b-3p-1 | -5.02 | miR-22-3p_miR-19b-3p-2 | 2.36 |
| miR-27a-3p_miR-122-5p | -0.47 | miR-101-3p-2_miR-19b-3p-1 | -3.03 |
| let-7f-5p-2_miR-146a-5p | -0.20 | miR-30d-5p_miR-20a-5p | 0.15 |
| miR-15b-5p_miR-16-5p-1 | -0.07 | let-7b-5p_miR-19b-3p-1 | -0.85 |
|  |  | miR-15a-5p_miR-16-5p-2 | -0.07 |
|  |  | miR-20a-5p_miR-19b-3p-1 | -2.08 |
|  |  | miR-21-5p_miR-23a-3p | -0.27 |

Table S.2 Spearman rank correlation analysis of the ratios between the two platforms

| \| Ratio \| Correlation  estimate \| p-value \| \| --- \| --- \| --- \| \| miR-26b-5p_miR-142-5p \| 0.3446 \| <0.0001 \| \| miR-101-3p_miR-19b-3p \| 0.2844 \| 0.0010 \| \| let-7b-5p_miR-19b-3p \| 0.2465 \| 0.0047 \| \| let-7f-5p_miR-19b-3p \| 0.2399 \| 0.0060 \| \| let-7a-5p_miR-320a \| 0.2387 \| 0.0062 \| \| miR-27a-3p_miR-122-5p \| 0.2346 \| 0.0072 \| \| miR-199a-3p_let-7a-5p \| 0.2273 \| 0.0093 \| \| miR-30d-5p_miR-20a-5p \| 0.1798 \| 0.0407 \| \| miR-93-5p_miR-19b-3p \| -0.1515 \| 0.0853 \| \| miR-20a-5p_miR-19b-3p \| 0.1507 \| 0.0870 \| \| miR-335-5p_let-7f-5p \| 0.1424 \| 0.1060 \| \| miR-22-3p_miR-19b-3p \| 0.1270 \| 0.1499 \| \| let-7f-5p_miR-146a-5p \| 0.1229 \| 0.1635 \| \| miR-15b-5p_miR-16-5p \| -0.1215 \| 0.1683 \| \| miR-21-5p_miR-23a-3p \| -0.1108 \| 0.2096 \| \| let-7a-5p_miR-106b-5p \| 0.0928 \| 0.2937 \| \| let-7a-5p_miR-22-3p \| 0.0848 \| 0.3372 \| \| let-7f-5p_miR-103 \| -0.0777 \| 0.3799 \| \| miR-199a-3p_let-7f-5p \| 0.0718 \| 0.4171 \| \| miR-15a-5p_miR-16-5p \| 0.0658 \| 0.4572 \| |
| --- | --- | --- | --- | --- | --- | --- | --- | --- | --- | --- | --- | --- | --- | --- | --- | --- | --- | --- | --- | --- | --- | --- | --- | --- | --- | --- | --- | --- | --- | --- | --- | --- | --- | --- | --- | --- | --- | --- | --- | --- | --- | --- | --- | --- | --- | --- | --- | --- | --- | --- | --- | --- | --- | --- | --- | --- | --- | --- | --- | --- | --- | --- | --- |
