# Supplementary material for: Plasma microRNA ratios associated with breast cancer detection in a nested case–control study from a mammography screening cohort
Source: Sci Rep. 2023 Jul 25;13:12040. doi: 10.1038/s41598-023-38886-0 (PMC10368693; doi:10.1038/s41598-023-38886-0)
Supplement: Supplementary file 2 — Supplementary Figures. [file 41598_2023_38886_MOESM2_ESM.pdf]

A)

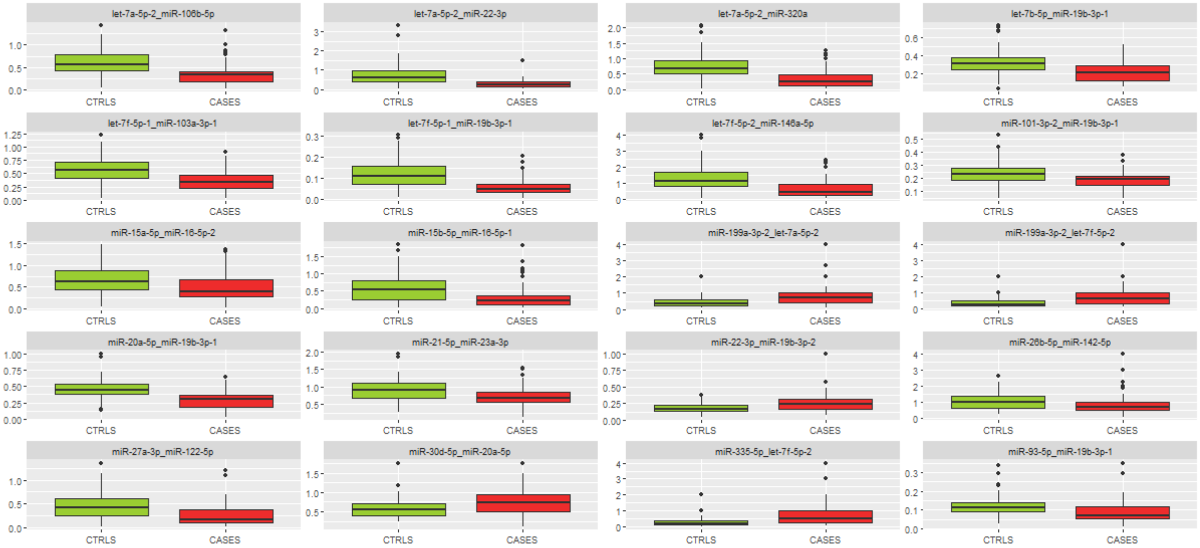

B)

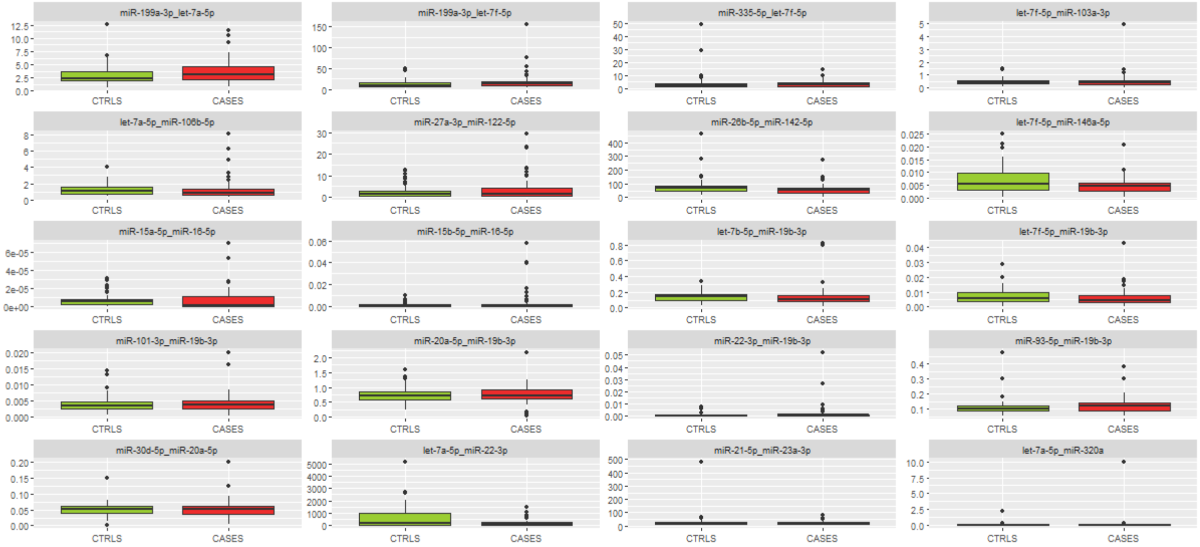

Figure S.1 Boxplot for miR ratios on small-RNA sequencing (panel A) and q-RT-PCR (panel B) data in cases (red) and controls (green). As ratios on sequencing data originally were not $\log_2$-transformed, to enable direct comparability of the results, RT-qPCR expression values were reported as 2^{Ct}

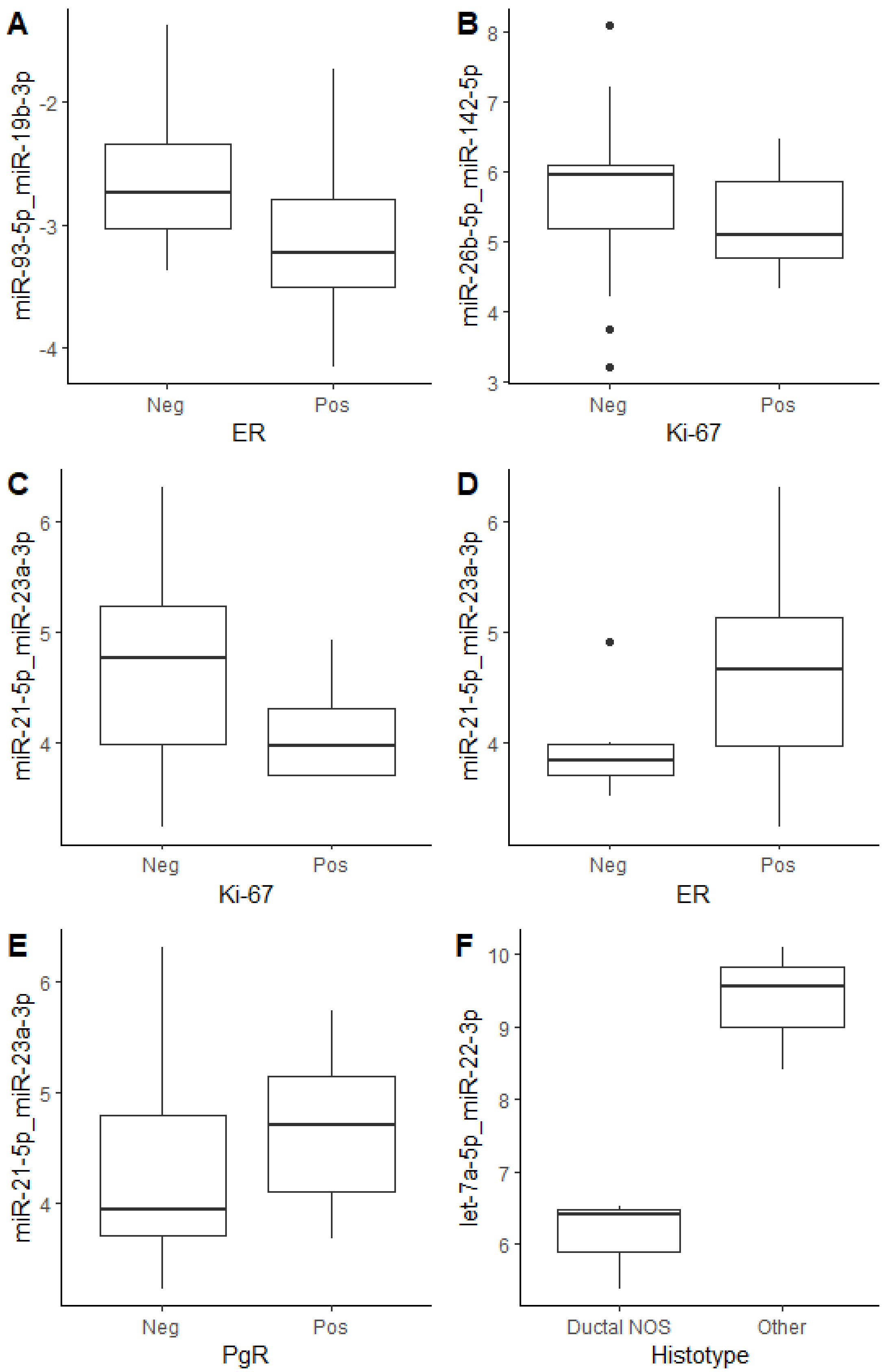

Figure S.2 Expression values for ratio associated with clinico-pathological BC cases characteristics. Panels A-E refer to invasive tumors whereas panel F to *in situ* ones

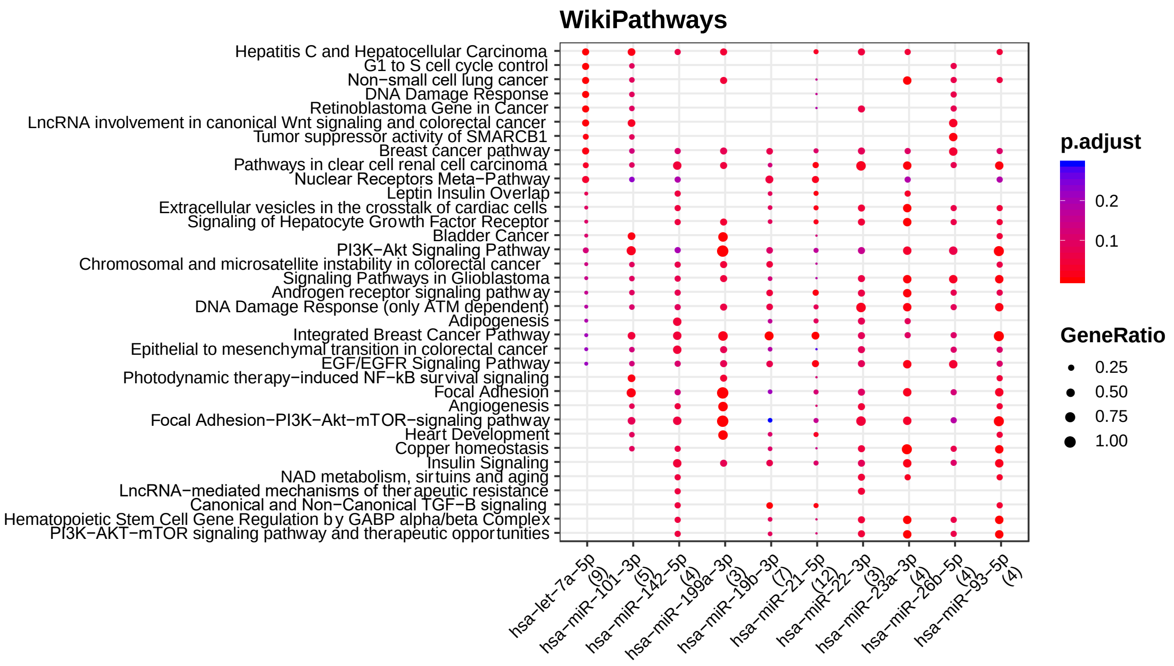

Figure S.3 Functional target enrichment results (Wikipathways database) on miRs making up the ratios within the model including non-molecular variables
